# A Novel Cross Injection Analysis for Simultaneous Multi-Determination of Diabetic Nephropathy Biomarkers in Urine

**DOI:** 10.3390/molecules31101772

**Published:** 2026-05-21

**Authors:** Prawpan Inpota, Nathawut Choengchan

**Affiliations:** 1 Flow-Innovation Research for Science and Technology Laboratories (FIRST Labs), Department of Chemistry, School of Science, King Mongkut’s Institute of Technology Ladkrabang, Bangkok 10520, Thailand; praw.inpota@gmail.com; 2 Department of Chemistry and Applied Analytical Chemistry Research Unit, School of Science, King Mongkut’s Institute of Technology Ladkrabang, Bangkok 10520, Thailand

**Keywords:** cross injection analysis, flow-based technique, albumin, creatinine, glucose, urine

## Abstract

This work presents, for the first time, a novel cross injection analysis (CIA) system for the simultaneous multi-determination of key biomarkers associated with diabetic nephropathy—namely, albumin, creatinine, and glucose—within a single analytical run. Unlike conventional flow-based techniques that rely on sequential measurements, the proposed CIA platform integrates multiple analytical pathways into a unified design, enabling one-shot multi-analyte analysis without the need for complex separation units or injection valves. The system employs peristaltic pumps and a rectangular platform with orthogonal flow channels, allowing concurrent aspiration and efficient transport of reaction products to compact detectors. Albumin determination was based on ion-association with tetrabromophenolphthalein ethyl ester. Creatinine was measured using the Jaffé reaction. Glucose was colorimetrically detected via its reaction with 3,5-dinitrosalicylic acid. The developed CIA provides enhanced sensitivity through its pre-mixing effect, enabling reliable quantification of trace analytes. Excellent analytical performance was achieved, including wide linear ranges (*r*^2^ > 0.99), good precision (RSD < 7%), and rapid analysis (5 min). The method was validated against established reference methods, showing no significant differences, and successfully applied to urine with satisfactory recoveries (84.8–107.3%). Importantly, the proposed system adheres to green chemistry by minimizing reagent consumption and waste generation, offering a sustainable approach for multi-parameter clinical analysis.

## 1. Introduction

Diabetic nephropathy is a progressive kidney disease resulting from long-term diabetes and is currently the leading cause of kidney failure. It is estimated that nearly one in five individuals with diabetes will require treatment for diabetic nephropathy [1]. The determination of key urinary biomarkers—namely, albumin, creatinine, and glucose—provides a valuable approach for clinical diagnosis and disease monitoring. Elevated urinary albumin levels above 30 mg L^−1^ are considered an early indicator of kidney dysfunction [1]. Creatinine is excreted into urine via glomerular filtration at a relatively constant rate and is commonly used as a reference marker for renal function [2]. Its minimum value has been set at 50 mg L^−1^ [3], and a greater excreted level indicates kidney disorder. Under normal physiological conditions, glucose is not present in urine, as it is almost completely reabsorbed in the renal tubules. The presence of glucose in urine (glycosuria) typically reflects renal impairment or uncontrolled blood glucose levels. The normal urinary glucose level should not exceed approximately 14.4 mg dL^−1^ [4].

A considerable number of studies have been reported on the determination of these biomarkers in urine and other biological fluids. For albumin, various analytical techniques have been developed, including colorimetric [5,6], fluorometric [7,8], and chromatographic methods [9,10]. Creatinine analysis has been widely performed using the traditional Jaffé reaction [11], enzymatic assays [12], and separation techniques [13,14,15,16]. For glucose determination, numerous modern approaches have been introduced, such as spectrophotometric, electrochemical, luminescence-based, surface-enhanced Raman spectroscopic, and enzymatic methods [17,18]. Although these methods provide reliable and accurate results, most are conducted in a batchwise manner, which is labor-intensive and time-consuming. In addition, batch analysis typically requires relatively large volumes of reagents and generates substantial chemical waste, raising concerns regarding environmental impact and sustainability. More importantly, these methods are generally designed for the determination of a single biomarker, limiting their practicality for routine analysis where multiple analytes must be measured simultaneously in a large number of urine samples.

Recently, Kiwfo et al. [19] demonstrated the simultaneous determination of albumin, creatinine, and glucose using a sequential injection–lab-at-valve (SI-LAV) approach [20,21,22]. The SI-LAV system was constructed using a multi-port selection valve and a high-precision syringe pump for automated liquid handling under computer control. While the proposed method is robust and demonstrates excellent potential for system miniaturization, the sequential operation of the SI technique may moderately limit analytical speed, since samples and reagents are aspirated in a stepwise manner. Consequently, this restricts sample throughput. Therefore, there is a clear need to develop an alternative flow-based technique that enables true simultaneous aspiration of samples and reagents, allowing multi-analyte determination with improved sample throughput.

In 2013, Nacapricha et al. [23] introduced a novel flow-based strategy termed cross injection analysis (CIA). The CIA platform consists of a main analytical flow channel (x-axis) intersected perpendicularly by multiple auxiliary channels (y-axis), all fabricated within a rectangular acrylic block. The y-axis channels are connected via tubing to a multi-channel peristaltic pump, enabling the simultaneous introduction of small volumes of sample and reagents into the x-axis channel through individual pump lines. In contrast to SIA systems, CIA allows the concurrent aspiration of sample and reagents, significantly enhancing operational efficiency. Notably, the system requires only a multi-channel peristaltic pump for liquid handling, eliminating the need for multi-port selection valves or syringe pumps. CIA has been successfully applied to a variety of analytical applications [23,24,25], as summarized in Table S1 (Section S1). However, previous studies have primarily demonstrated CIA for single- or dual-analyte determination. To the best of our knowledge, no study has yet reported the simultaneous determination of multiple analytes using a CIA platform.

Therefore, this work aims, for the first time, to develop a novel CIA method for simultaneous multi-analyte determination. The proposed system was applied for the measurement of albumin, creatinine, and glucose as a proof of concept. Three CIA platforms were interconnected via a urine introduction conduit (see Section 2.1), enabling integrated and parallel analysis within a single system. All liquid handling procedures were fully automated and controlled using in-house developed software. The analytes were detected simultaneously using three individual detectors operating in parallel. Albumin was determined via an ion-association reaction with tetrabromophenolphthalein ethyl ester (TBPE), which provides higher molar absorptivity than conventional dyes [26]. Creatinine detection was based on the well-established colorimetric Jaffé reaction [11], while glucose was determined using a simple non-enzymatic assay with 3,5-dinitrosalicylic acid (DNS), producing a red-colored product for spectrophotometric detection [27]. Applicability of the proposed CIA method to human urine samples associated with complex matrices was demonstrated. Comprehensive validation studies, including accuracy, precision, and method comparison with reference techniques, were also performed.

## 2. Results and Discussion

### 2.1. Manifold Design

In our previous work [24], a CIA-based method was developed for the dual determination of Fe^3+^ and creatinine in urine samples from thalassemic patients undergoing medication. In that configuration, two CIA platforms were employed for the independent introduction and mixing of the sample and reagents and were arranged in tandem to generate discrete reaction zones. The y-channels of each platform were used for loading the sample and reagents, while the x-channels were connected to mixing coils to form the analytical flow path toward the detection cell. A single diode-array spectrophotometer was used for sequential monitoring of the resulting product zones. To avoid overlap between consecutive product plugs, a delay coil was required between the platforms; however, this additional component led to increased analysis time and reduced sample throughput.

**Figure 1:**
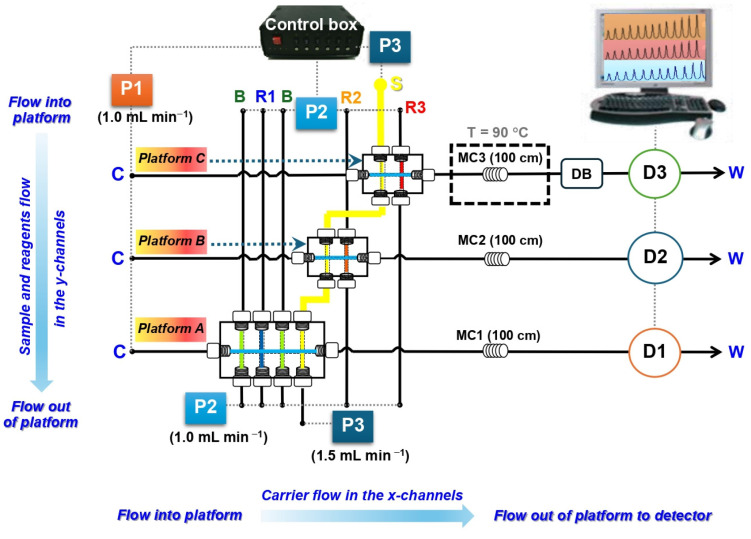
A simplified schematic diagram of the developed CIA manifold for the simultaneous multi-determination of albumin (platform A), creatinine (platform B), and glucose (platform C) in urine. P: peristaltic pump; MC: mixing coil; DB: de-bubbler unit; D1: spectrophotometer (600 nm); D2–D3: green-LED detectors; C: carrier (water); S: mixed standards/urine samples (yellow line); R1: TBPE (2 × 10^−4^ mol L^−1^) with Triton X-100 (0.2% (*v*/*v*)); B: 0.01 mol L^−1^ acetate buffer (pH 3.2); R2: alkaline picrate (0.025 mol L^−1^ in 0.2 mol L^−1^ NaOH); R3: 2.0% (*w*/*v*) DNS; W: waste. All peristaltic pumps were fully automated via in-house software and housed in a control box. Pale blue arrows (aligned vertically and horizontally) indicate the flow directions of the liquids.

To address this limitation, the present work aims to develop an improved CIA manifold enabling faster multi-analyte determination without the need for delay coils. A simplified schematic of the proposed manifold is shown in Figure 1. The system consists of three independent CIA platforms, designated as A, B, and C, for the determination of albumin, creatinine, and glucose, respectively. Note that the platforms were fabricated from transparent acrylic material, similar to that reported in previous studies [23,24,25]. Detailed dimensions of the CIA platforms are provided in Figure S1 (Section S2).

In contrast to the previous design [24], the present manifold was constructed by interconnecting the platforms through the sample introduction conduits (depicted as the yellow line in Figure 1), rather than arranging them in series along the main flow path. This configuration enables the use of three independent detectors for parallel analysis of the target biomarkers. The peristaltic pump P1 was employed to deliver the carrier solution (C) through the x-axis channels, while pumps P2 and P3 were used for the simultaneous filling and withdrawal of the respective reagents (TBPE, buffer, alkaline picrate, and DNS) and the sample/standard solutions (S), respectively. Three separate detectors, designated as D1, D2, and D3, were implemented for the parallel monitoring of the corresponding product zones.

It should be noted that the sequence of analytical platforms in the proposed manifold was intentionally designed as follows: glucose determination (platform C) → creatinine determination (platform B) → albumin determination (platform A). The arrangement was primarily selected to minimize possible backflow or carryover of reagent solutions into the common sample channel connected to subsequent analytical platforms. In particular, the albumin determination system employed TBPE and Triton X-100 as reaction reagents. Due to the configuration of the albumin platform, the sample inlet was positioned at the outermost channel of the cross-injection interface, allowing the reagent stream to potentially turn and partially diffuse into the sample conduit during operation. If the albumin platform were positioned upstream, traces of TBPE/Triton X-100 could subsequently contaminate downstream platforms and interfere with other determinations. Therefore, the albumin platform was placed as the last platform in the manifold sequence to prevent reagent contamination of subsequent analytical systems. In contrast, for the glucose and creatinine platforms, the sample stream entered the cross-injection interface prior to the reagent streams, significantly reducing the possibility of reagent backflow into the sample channel. The glucose platform was additionally positioned as the first platform because this determination required a thermostatic water bath for the colorimetric reaction. Locating this platform at the beginning of the manifold simplified the overall system configuration and improved operational convenience.

The analytical workflow was initiated by simultaneously activating all peristaltic pumps. The sample stream (yellow line in Figure 1) was sequentially introduced into the sample conduits of platforms C, B, and A, while the corresponding reagents were simultaneously delivered into each platform. Under continuous carrier flow, the sample and reagent zones were merged and pre-mixed in situ, which has been shown to enhance analytical sensitivity [23,24]. As the carrier stream continuously flowed while the sample was propelled through all CIA platforms in the proposed manifold, dilution effects were expected to be minimal due to the short distance between adjacent platforms (~30 mm), narrow tubing dimensions (1.0 mm i.d.), and continuous propulsion of the sample stream at a constant flow rate (1.0 mL min^−1^). Furthermore, while the carrier stream continuously flowed, the sample and reagent solutions were simultaneously impelled toward each CIA platform. Under these conditions, controlled hydrodynamic dispersion and partial pre-mixing among the aspirated zones occurred before the merged zones subsequently passed through the mixing coil. This pre-mixing phenomenon was not considered a detrimental dilution effect; instead, it promoted better zone homogenization and enhanced interaction between the analyte and reagents. Such a characteristic significantly enhances the capability for accurate trace quantitative analysis. The resulting reaction products were then transported along the x-axis channels toward the flow cells positioned within each detector. The corresponding CIA signal profiles were recorded and displayed on a single monitoring interface. With this parallel configuration, the need for a delay coil is eliminated, thereby significantly reducing analysis time and increasing sample throughput. This improvement is particularly advantageous for routine analytical applications. In addition, all liquid handling operations were automatically controlled using in-house developed software, providing a user-friendly and fully programmable system.

### 2.2. Optimization Study

The key parameters influencing the analytical sensitivity and sample throughput of the developed CIA method (Figure 1) were systematically optimized using a univariable (one-factor-at-a-time) approach. The effect of each parameter is discussed in the following sections.

#### 2.2.1. For the Albumin Determination (Platform A)

The determination of albumin was based on ion-association with tetrabromophenolphthalein ethyl ester (TBPE), which provides higher molar absorptivity than other organic dyes [26]. A blue-colored complex is formed, with a maximum absorption wavelength (λ_max_) at 600 nm (Figure S2A, Section S3).

Initially, a handheld, red-LED detector was connected to the outlet of platform A to monitor the flowing product zone. However, the obtained sensitivity was insufficient for reliable quantification. This limitation is likely attributed to the spectral mismatch between the LED emission and the absorption maximum of the complex. To overcome this drawback, a compact spectrophotometer was employed instead, allowing wavelength-specific detection and significantly improved sensitivity.

For the optimization of albumin determination, the concentrations of the chromogenic reagents were adopted from a previous study [28], while the physical parameters of the CIA system were systematically investigated to maximize analytical performance. In particular, the influence of flow configuration on zone interaction and reaction efficiency was critically evaluated.

**Figure 2:**
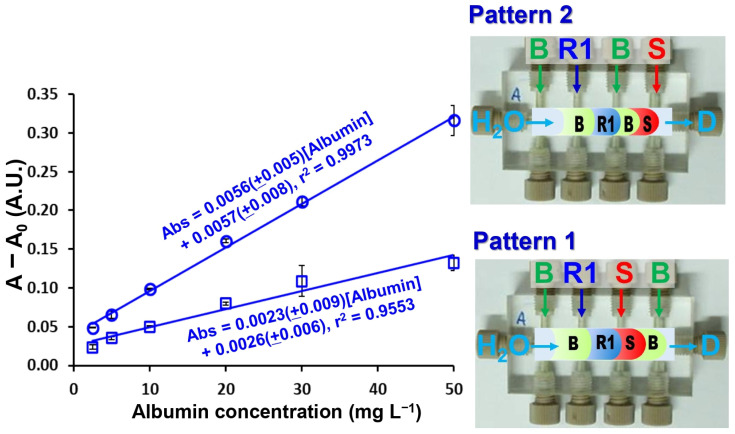
Effect of aspirated zone sequences of the sample and reagents on the sensitivity of albumin determination in platform A. S: albumin standard (2.5–50 mg L^−1^); R1: TBPE (2 × 10^−4^ mol L^−1^) with Triton X-100 (0.2% (*v*/*v*)); and, B: 0.01 mol L^−1^ acetate buffer (pH 3.2).

Firstly, the aspiration sequence of the sample and reagents into platform A was examined. In pattern 1, the sample and reagent (TBPE, R1 in Figure 2) zones are confined between two buffer zones (B in Figure 2); in contrast, in pattern 2, the buffer zones are premixed with the TBPE zone prior to interaction with the sample. As shown in Figure 2, pattern 2 yielded significantly higher sensitivity, as reflected by a steeper calibration slope, along with improved linearity. This enhancement can be attributed to more effective pre-mixing between the TBPE reagent and the buffer solution, which plays a crucial role in activating the chromogenic system. This configuration also promotes sufficient protonation equilibrium and micelle formation before exposure to albumin, leading to more efficient binding and signal generation. According to Sakai et al. [26], TBPE must first be protonated under acidic conditions to generate the reactive species (TBPE·H). This protonated form is subsequently incorporated into the micellar environment provided by Triton X-100, which enhances its hydrophobic interaction and facilitates subsequent association with albumin. Therefore, the extent of TBPE protonation and micellar stabilization directly governs the efficiency of complex formation and, consequently, the analytical sensitivity. In contrast, in less favorable sequences, incomplete protonation and/or insufficient micellar organization may occur, resulting in reduced interaction between TBPE and albumin and thus lower sensitivity.

From a hydrodynamic perspective, the improved performance of pattern 2 may also be associated with enhanced zone interpenetration and reduced axial dispersion mismatch between reagent and buffer zones, allowing more homogeneous reaction conditions to be established before detection. Consequently, pattern 2 was selected as the optimal configuration for subsequent studies.

The effect of the mixing coil length (MC1, Figure 1) on analytical performance was subsequently investigated over the range of 50–200 cm using two albumin standard solutions (10 and 50 mg L^−1^). As shown in Figure S3A (Section S3), no significant difference in signal intensity was observed at 10 mg L^−1^ across the studied range, suggesting that mixing was already sufficient for complete reaction at this concentration. In contrast, at 50 mg L^−1^, the signal intensity increased as the coil length was extended from 50 to 100 cm, indicating that improved mixing and longer residence time enhanced the extent of complex formation. This behavior can be attributed to more effective interpenetration of the reagent and sample zones, allowing the reaction between albumin and the activated TBPE species to proceed more completely. However, further increasing the coil length beyond 100 cm resulted in a decrease in signal intensity. This decline is primarily because of excessive axial dispersion and dilution of the reaction zone, which reduces the peak concentration of the colored product reaching the detector. Such dispersion effects become more pronounced at longer coil lengths, ultimately compromising sensitivity. Since a coil length of 100 cm provided the highest signal intensity while maintaining efficient mixing, it was selected as the optimal condition.

#### 2.2.2. For the Creatinine Determination (Platform B)

The determination of creatinine relies on the well-established Jaffe reaction [11], in which creatinine reacts with alkaline picrate (picric acid in sodium hydroxide) to form a stable, orange-colored complex. This chromogenic product exhibits a maximum absorption wavelength (λ_max_) at 480 nm, enabling its quantitative determination by colorimetric detection (Figure S2B, Section S3). In this work, a green-LED detector (D2) was employed for the colorimetric measurement of the developed product (Figure 1). The reagent concentrations were adopted from a previous study [29], and only the physical parameters of the CIA system were optimized.

The effect of the mixing coil length (MC2, Figure 1) was explored over the range of 50–200 cm using creatinine standard solutions of 100 and 500 mg L^−1^. As shown in Figure S3B (Section S3), increasing the coil length from 50 to 100 cm resulted in a pronounced increase in absorbance, indicating enhanced reaction efficiency. This improvement can be attributed to extended residence time and more effective interpenetration of the sample and reagent zones, which facilitates the formation of the creatinine–picrate complex under flow conditions.

Beyond 100 cm, only a marginal increase in absorbance was observed, while the analysis time increased noticeably. This behavior indicates that the reaction had approached completion, and further extension of the coil primarily induced axial dispersion, leading to dilution of the reaction zone and diminishing returns in sensitivity. Therefore, a coil length of 100 cm was selected as an optimal compromise between sensitivity and sample throughput. It is also noteworthy that the aspiration sequence had no significant effect on the analytical signal. This suggests that, unlike the albumin–TBPE system, the Jaffe reaction is less dependent on pre-equilibration or specific zone ordering.

#### 2.2.3. For the Glucose Determination (Platform C)

In this work, a non-enzymatic approach based on the colorimetric reaction between glucose and 3,5-dinitrosalicylic acid (DNS) was employed due to its simplicity and robustness. Although enzymatic methods based on glucose oxidase are widely used owing to their high sensitivity and selectivity, their practical application is often limited by poor long-term operational stability, limited reusability, and restricted shelf-life of the enzyme. In addition, the relatively high cost of enzymes further constrains their applicability, particularly in resource-limited or routine analytical settings. From a green analytical chemistry perspective, the enzyme-free approach also reduces the need for biologically derived reagents and minimizes storage constraints.

Based on the glucose–DNS reaction, a red-colored product is formed, exhibiting λ_max_ at 510 nm (Figure S2C, Section S3). In this work, a green-LED detector (D3) was employed to monitor the flowing product zone from platform C (Figure 1). Both chemical and physical parameters affecting sensitivity and analysis time were systematically optimized using glucose standard solutions of 10 and 50 mg dL^−1^. Initially, the effect of the mixing coil length (MC3, Figure 1) was evaluated over the range of 50–200 cm using glucose standard solutions of 10 and 50 mg dL^−1^. The results in Figure S3C (Section S3) show that the coil length had no significant effect on the sensitivity for glucose determination at 10 mg dL^−1^. At 50 mg dL^−1^, the signal increased as the length was extended from 50 to 100 cm. Nonetheless, a further increase in length resulted in a decrease in signal, likely due to greater dilution. Therefore, a coil length of 100 cm was considered optimal.

The effect of DNS concentration was investigated over the range of 0.5–3.0% (*w*/*v*). The highest signal intensity was observed at 2.0% (*w*/*v*) (Figure S4A, Section S3), which was therefore selected as the optimal concentration. This behavior implies that an adequate excess of DNS is required to drive the redox reaction toward completion, while excessive reagent concentrations may increase background absorbance and reduce the signal-to-noise ratio.

Since the DNS–glucose reaction is thermally accelerated, the effect of incubation temperature was further examined by immersing the mixing coil (MC3, Figure 1) in a temperature-controlled bath. At a lower glucose concentration (10 mg dL^−1^), temperature showed no significant effect on signal intensity. In contrast, at a higher concentration (50 mg dL^−1^), the absorbance increased markedly as the temperature was raised from 85 to 90 °C (Figure S4B, Section S3), reflecting enhanced reaction kinetics and more efficient conversion of DNS to the reduced product (3-amino-5-nitrosalicylic acid).

However, operation at elevated temperatures led to the formation of air bubbles within the flow system, which were trapped in both the conduits and the flow cell, thereby causing signal instability and baseline fluctuation. To address this issue, a debubbler unit (DB, Figure 1) was incorporated into the system. This unit, adapted from a Metrohm^TM^ gas-diffusion module (model 754, Switzerland), was equipped with a 0.45 μm pore-size circular PTFE membrane (47 mm i.d., 0.8 mm thickness, Sartorius, Germany) to selectively remove gaseous components from the flowing liquid stream via passive diffusion. The integration of this unit effectively minimized bubble-related disturbances and improved signal stability and reproducibility.

### 2.3. Analytical Features

**Figure 3:**
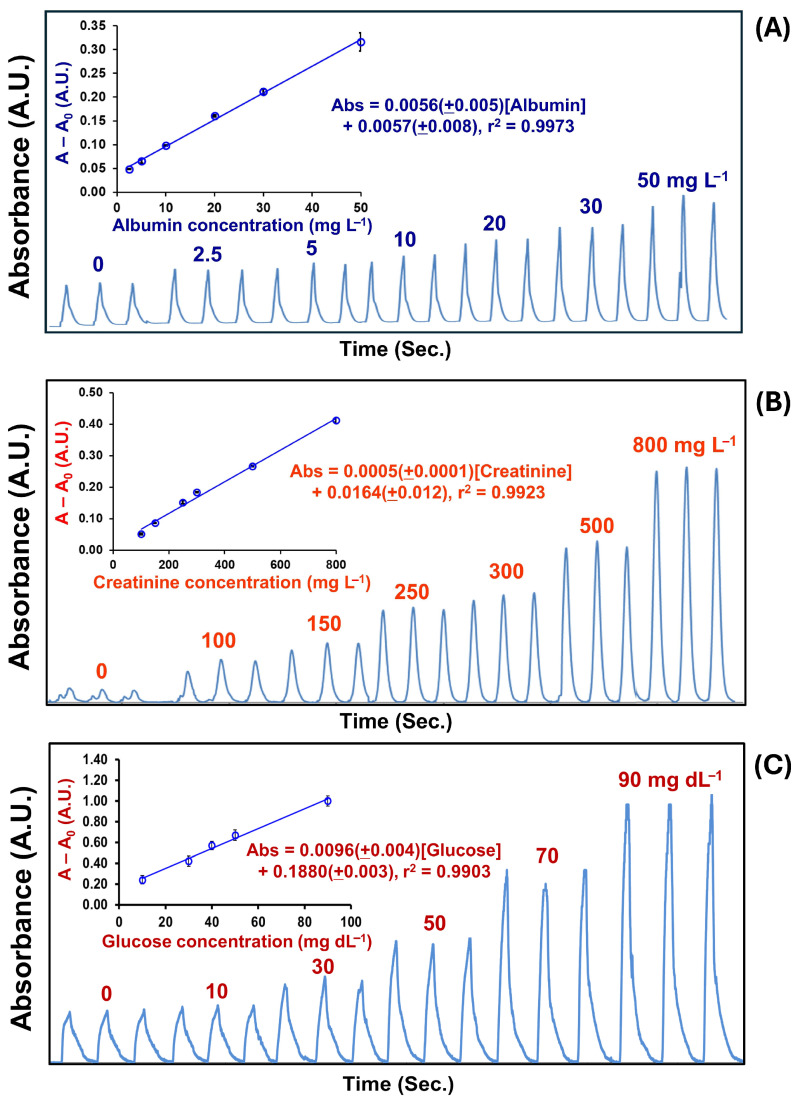
Representative signal profiles and corresponding calibration plots with regression equations obtained using the developed CIA system for the simultaneous multi-determination of (**A**) albumin (0–50 mg L^−1^), (**B**) creatinine (0–800 mg L^−1^), and (**C**) glucose (0–90 mg dL^−1^). The y-axis of the calibrations (A − A_0_) represents the difference between the absorbance of the product and the blank.

Under the optimized conditions, the analytical performance of the developed CIA system for the simultaneous multi-determination of albumin, creatinine, and glucose is summarized in Table 1. Representative CIA signal profiles and their corresponding calibration curves are presented in Figure 3A–C. Analytical signals were processed by subtracting the blank signal prior to quantification. Calibration curves were constructed using standard solutions over the concentration range of 2.5–50 mg L^−1^ albumin, 100–800 mg L^−1^ creatinine, and 10–90 mg dL^−1^ glucose. Linear regression analysis [30] was applied to obtain the calibration equations. The system exhibited wide linear working ranges for all analytes, with good linearity (*r*^2^ > 0.99), satisfactory precision (RSD < 7%), and highly reproducible signal profiles.

The limit of detection (LOD) values—calculated according to LOD = y_B_ + 3S_B_ where y_B_ and S_B_ denote the intercept and the random error in the y-direction of the calibration curve, respectively [30]—were sufficiently low for practical application in the monitoring of diabetic nephropathy. Notably, the clinically relevant threshold concentrations in urine for early-stage diabetic nephropathy are approximately 30 mg L^−1^ for albumin [1], 50 mg L^−1^ for creatinine [3], and 14.4 mg dL^−1^ for glucose [4]. The achieved detection limits of the proposed method are well within these clinically significant ranges, highlighting its suitability for early screening and routine monitoring.

A key advantage of the developed CIA system lies in its operational simplicity and integration capability. The CIA manipulation enables the simultaneous introduction of the sample and all reagents into the flow system within a single aspiration step. The manifold was designed as a unified platform that supports parallel detection of three biomarkers in a single analytical run. Consequently, the complete multi-analyte determination can be accomplished within 5 min. This configuration offers a significant advantage over sequential injection (SI) systems, where samples and reagents must be aspirated sequentially, resulting in more complex operation and longer analysis time. In addition, the fabrication of the CIA platforms from acrylic material provides a cost-effective alternative to electronically controlled multi-selection valves typically employed in SI systems. The simplicity of the design also reduces maintenance requirements and enhances system robustness for routine use.

Compared to our previously reported CIA method for dual-analyte determination [24], the sample throughput achieved for triple-analyte analysis in this work was improved, increasing from 10 to 12 samples h^−1^. Although the increase is modest, it reflects enhanced analytical efficiency resulting from the simplified manifold design. This enhancement is primarily attributed to the elimination of the delay coil required in the earlier serial configuration, as the present manifold operates in a parallel format. The absence of a delay coil not only shortens the analysis time but also minimizes dispersion-related signal broadening, thereby contributing to improved analytical efficiency.

From an application standpoint, the combined analytical figures of merit and operational advantages demonstrate that the proposed CIA system represents a practical and powerful strategy for routine multi-analyte monitoring in clinical samples. Furthermore, the method aligns well with the principles of green analytical chemistry, as it minimizes reagent consumption, reduces sample volume, and limits waste generation through integrated, simultaneous analysis, while maintaining reliable performance for accurate quantification in complex urine matrices.

### 2.4. Application to Urine Samples: Recovery Study and Validation

To verify the applicability of the developed CIA method, it was applied to discarded anonymous human urine samples. The analyte concentrations in samples were calculated based on the obtained calibration equation. All measurements were performed in triplicate and expressed as mean ± standard deviation. The analytical recovery of the method was first evaluated. The samples, designated as S1 and S2 in Table 2, were filtered through a 0.22 µm nylon membrane filter, and the filtrates were subsequently diluted with water at appropriate dilution ratios. The diluted samples were then spiked with standard solutions to obtain final concentrations of 30 mg L^−1^ albumin, 100 mg L^−1^ creatinine, and 30 mg dL^−1^ glucose. As shown in Table 2, the recoveries ranged from 84.8% to 107.3%. The overall recovery range falls within generally accepted criteria for bioanalytical methods, confirming the reliability of the proposed method. These results confirm that the simultaneous determination of all three biomarkers using the developed CIA system is not significantly affected by matrix components in diluted urine samples. The dilution step likely plays a key role in minimizing potential interferences by reducing matrix complexity. In addition, the flow-based nature of the CIA system, which involves continuous dilution and dispersion of the sample zone, may further mitigate matrix-induced signal suppression.

From an analytical perspective, the satisfactory recoveries also indicate good selectivity of the employed chemistries (TBPE ion-association, Jaffe reaction, and DNS reaction) under the optimized conditions. Importantly, the absence of significant cross-interference among the three analytes within the integrated manifold highlights the effectiveness of the platform design for multi-analyte detection.

Potential interference in urine analysis may arise from endogenous compounds and metabolites present in the complex sample matrix. For albumin determination based on the TBPE reaction, interference may originate from other positively charged species capable of interacting with the dye reagent. For creatinine determination using the alkaline picrate reaction, reducing substances and carbonyl-containing compounds may contribute to non-specific Jaffé-type reactions. For glucose determination based on the reaction with 3,5-dinitrosalicylic acid (DNS), other reducing compounds such as ascorbic acid and uric acid may also react with the reagent. Although a comprehensive investigation of individual interfering species was not carried out in this study, the reliability of the proposed method was supported by recovery studies using real urine samples, which demonstrated acceptable analytical accuracy and can be considered reliable for the diabetic nephropathy biomarkers determination in urine samples. Therefore, the current data are considered adequate to validate the applicability of the proposed method.

**Figure 4:**
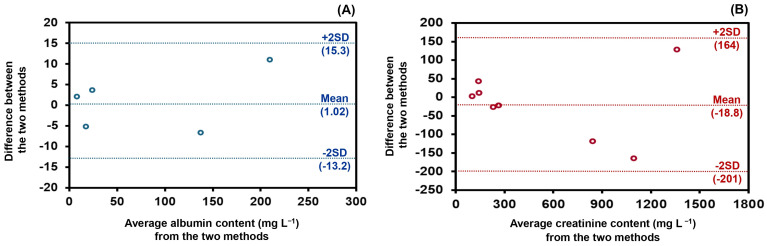
Validation based on Bland–Altman plot for comparison of results obtained from the developed CIA method and conventional batchwise spectrophotometric methods for the determination of (**A**) albumin (*n* = 5; samples S3–S7 in Table S2, Section S4) and (**B**) creatinine (*n* = 8; samples S3–S10 in Table S2, Section S4).

Secondly, the concentrations of the target biomarkers in urine samples were determined, and the results are summarized in Table S2 (Section S4). The urinary albumin and creatinine contents obtained by the developed CIA method were statistically compared with those obtained using conventional batchwise spectrophotometric methods based on the same reaction principles. The agreement between the two methods was evaluated using the Bland–Altman difference plot [31]. As shown in Figure 4A,B, all values fall within ± 2 SD of the mean difference, indicating good agreement and the absence of significant systematic bias between the methods. Moreover, statistical significance was assessed using the paired *t*-test [30]. The results demonstrated good agreement at the 95% confidence level (albumin: *t*_stat_ = 2.32, *t*_critical_ = 2.78, d.f. = 4; creatinine: *t*_stat_ = 0.56, *t*_critical_ = 2.20, d.f. = 7), confirming that there is no significant difference between the proposed and reference methods. These indicate that the developed CIA system provides comparable accuracy to established batchwise techniques.

For glucose determination, validation was performed using a commercial urine glucose test strip. The results obtained from the CIA method were compared with those from the test strip using the *t*-test approach [30]. No statistically significant difference was observed at the 95% confidence level (*t*_stat_ = 4.15, *t*_critical_ = 4.30, d.f. = 2), indicating acceptable agreement between the two methods. Despite the difference in detection principles (DNS-based vs. enzymatic strip), the observed agreement highlights the robustness of the non-enzymatic approach.

These results confirm that the developed CIA method provides high accuracy, good agreement with reference methods, and reliable performance for practical applications in real sample analysis.

Potential carryover or residual reagents in the common sample line may affect the sample composition, particularly in a multi-platform CIA system. In the present study, direct evaluation of such effects using a before–after control experiment was not performed. However, the possibility of cross-interference was indirectly assessed through recovery experiments using real urine samples, which showed acceptable recoveries (84.8–107.3%), suggesting that no significant cumulative interference occurred under the proposed operating conditions. In addition, the system was designed with continuous flow and sufficient washing steps between measurements to effectively minimize sample and reagent carryover within the common channel. The reliability of the proposed CIA system was also evaluated by comparing the analytical results of albumin and creatinine with those obtained from conventional batch methods using identical reaction chemistries. Statistical analysis showed no significant difference between the two approaches. This agreement suggests that any potential carryover or cross-interference within the CIA system is negligible under the operating conditions used and does not significantly affect the analytical results.

## 3. Materials and Methods

### 3.1. Standard and Reagent Preparation

All reagents were of analytical reagent grade. Deionized distilled water purified using a Milli-Q system was used throughout. Stock standard solutions of albumin (1000 mg L^−1^), creatinine (1000 mg L^−1^), and glucose (1000 mg L^−1^) were prepared by dissolving accurately weighed 0.100 g portions of human serum albumin (HSA, Fluka, Buchs, Switzerland), creatinine (Sigma-Aldrich, St. Louis, MO, USA), and glucose (Sigma-Aldrich, St. Louis, MO, USA), respectively, in 100.00 mL of water. The albumin stock solution was stored at 4 °C to prevent degradation. Working mixed standard solutions were freshly prepared by appropriate dilution to obtain final concentration ranges of 2.5–50 mg L^−1^ for albumin, 100–800 mg L^−1^ for creatinine, and 10–90 mg dL^−1^ for glucose.

A stock solution of TBPE (1 × 10^−3^ mol L^−1^) was prepared by dissolving TBPE (Sigma-Aldrich, USA) in ethanol. The working solution (R1, Figure 1) was prepared to yield 2 × 10^−4^ mol L^−1^ TBPE in 0.2% (*v*/*v*) Triton X-100. The buffer solution (B, Figure 1) was prepared by mixing 0.1 mol L^−1^ sodium acetate (Rankem, India) with 0.1 mol L^−1^ acetic acid (Mallinckrodt, Bangkok, Thailand) and adjusting the pH to 3.2 using a Methrom^TM^ pH meter (Model 780, Herisau, Switzerland).

A stock picric acid solution (0.1 mol L^−1^) was prepared by dissolving approximately 2.3 g of sodium picrate (Merck, Rahway, NJ, USA) in water. The working picrate solution (R2, Figure 1) was prepared by mixing picric acid with sodium hydroxide (Rankem, New Delhi, India) to obtain a final composition of 0.025 mol L^−1^ picric acid in 0.2 mol L^−1^ sodium hydroxide.

A 2% (*w*/*v*) 3,5-Dinitro salicylic acid or DNS solution (R3, Figure 1) was freshly prepared by dissolving 1.0 g of DNS (Buchs, Switzerland), 0.25 g of phenol (Carlo Erba, Milan, Italy), 9.1 g of sodium potassium tartrate (Ajax Finechem, New Zealand), and 0.5 g of sodium hydroxide in water, followed by dilution to a final volume of 50.0 mL.

### 3.2. Assembly of the CIA Manifold

A simplified schematic of the developed CIA manifold for the simultaneous multi-determination of albumin, creatinine, and glucose in urine is shown in Figure 1. The manifold consists of three independent CIA platforms, designated as platforms A, B, and C. These platforms were fabricated from transparent acrylic materials, similar to those described in previous studies [23,24,25]. Optical images and dimensions of the platforms are provided in Figure S1 (Section S2).

Platforms A, B, and C in Figure 1 were used for the introduction and mixing of samples and reagents for the measurement of albumin, creatinine, and glucose, respectively. All internal conduits within the platforms had a uniform inner diameter of 1.0 mm, except for the sample channel, which had an inner diameter of 2.0 mm. The manifold was assembled using 1.0 mm i.d. PTFE tubing (Cole-Parmer™, Vernon Hills, IL, USA) for interconnections and for the mixing coils (MC1, MC2, and MC3).

An eight-channel Ismatec™ pump (model ISM843, Glattbrugg, Switzerland), designated as P1 in Figure 1, was used to deliver the water carrier (C) through the x-channels of the flow system. A twelve-peristaltic pump (Ismatec™ IPN-12, Glattbrugg, Switzerland), designated as P2, was employed for the simultaneous filling and withdrawal of all reagents (R1, R2, R3, and B) through the y-channels of the platforms. A second Ismatec™ pump (same model as P1), designated as P3, was used for the simultaneous filling and withdrawal of the sample or mixed standard solutions (S) in the y-channels (indicated by the yellow line in Figure 1) across all platforms.

For platform A (albumin determination), the x-direction flow path was connected to a PerkinElmer™ 10 mm path-length flow-through cell (18 μL), installed in a JASCO™ UV–Vis spectrophotometer (Model 630, Easton, MD, USA), designated as D1 in Figure 1, for monitoring the reaction product at 600 nm. The flow lines of platforms B and C were connected to two separate green-LED detectors (Bangkok High Lab™, Thailand), designated as D2 and D3, for the determination of creatinine and glucose, respectively. Flow cells identical to that used in D1 were employed for these detections. A thermostated water bath (Fisher Scientific™, Isotemp 205, Pittsburgh, PA, USA) was used to heat the mixing coil MC3 to enhance the analytical sensitivity of glucose determination.

### 3.3. Steps of the Analytical Workflow

An in-house electronic control board (Figure 1) was used to automatically start and stop the peristaltic pumps (P1, P2, and P3). The system was operated via custom software written in Visual Basic 6.0 in combination with a programmable logic controller (PLC) for precise liquid handling. Details of the pump control program operation are provided in Section S5 of the Supplementary Materials. The analytical workflow for the simultaneous multi-determination of the three biomarkers is summarized in Table 3.

Briefly, in step 1, pump P1 was activated to deliver the water carrier through the x-channels of all platforms toward the detectors and subsequently to waste (W). In Step 2, all pumps (P1, P2, and P3) were switched on to simultaneously introduce the sample and corresponding reagents into each platform. This step contributes pre-mixing of sample plugs with reagent plugs which can enhance sensitivity [23]. In Step 3, pumps P2 and P3 were switched off while P1 remained in operation, allowing the reacted zones to be transported through the mixing coils for further mixing and subsequent detection. Prior to analyzing a new sample, all pumps were switched off and the sample aspiration tube was manually transferred from the previous sample solution to water. Finally, in Step 4, pumps P1 and P3 were activated while P2 was switched off to flush the flow channels with water, thereby preparing the system for the next analytical cycle. Specifically, the system operates under continuous flow conditions, and a washing step using deionized water was applied between successive samples (Step 4, Table 3). The flushing was carried out using a volume of 0.75 mL for a duration of 30 s, which is expected to effectively reduce residual sample within the common line (inner diameter 2.0 mm).

## 4. Conclusions

This work presents a novel CIA system for the simultaneous determination of multiple analytes (triple-analyte analysis) within a single-run analysis for the first time. The method was successfully demonstrated for the determination of key diabetic nephropathy biomarkers—albumin, creatinine, and glucose—in human urine. The system integrates three CIA platforms via a common sample introduction conduit, enabling parallel detection using individual detectors within a single analytical cycle. All liquid handling steps were fully automated using in-house developed software.

Under optimized conditions, the method provided good linearity (*r*^2^ > 0.99), high precision (RSD < 7%), and satisfactory recoveries (mean (±SD): 92% (±7)), with complete analysis achieved within 5 min. The method was successfully applied to real urine samples and validated against reference methods, confirming its reliability for complex matrices.

The developed system also aligns with green analytical chemistry principles by minimizing reagent consumption, reducing waste, and avoiding enzyme-based reagents. These features make the proposed CIA method a practical and sustainable strategy for the detection of trace biomarkers in complex biological matrices.

## Figures and Tables

**Table 1:** Summary of the analytical features achieved by the developed CIA approach for the simultaneous multi-determination of albumin, creatinine, and glucose.

Analytical Features	Diabetic Nephropathy Biomarkers		
	Albumin	Creatinine	Glucose
Working range	2.5–50 mg L^−1^	100–800 mg L^−1^	10–90 mg dL^−1^
Calibration and correlation coefficient (*r*^2^)	Abs = 0.0056(±0.005) [Albumin] + 0.0057(±0.008), *r*^2^ = 0.9973	Abs = 0.0005(±0.0001) [Creatinine] + 0.0164(±0.012), *r*^2^ = 0.9923	Abs = 0.0096(±0.004) [Glucose] + 0.1880(±0.003), *r*^2^ = 0.9903
RSD (*n* = 5)	3.4% (at 20 mg L^−1^)	5.1% (at 300 mg L^−1^)	6.7% (at 30 mg dL^−1^)
LOD (y_B_ + 3S_B_)	1.9 mg L^−1^	22.9 mg L^−1^	5.1 mg dL^−1^
Reagents consumption in platform (analysis^−1^)	TBPE: 23.6 µL Buffer: 47.2 µL	Alkaline picrate: 23.6 µL	DNS: 23.6 µL
Throughput	12 Samples h^−1^ (with a single run measurement of three analytes) 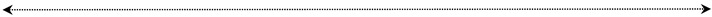		

Note: y_B_; intercept, S_B_; random error in the y-direction of the calibration curve, TBPE; tetrabromophenolphthalein ethyl ester (2 × 10^−4^ mol L^−1^) with Triton X-100 (0.2% (*v*/*v*)), buffer; 0.1 mol L^−1^ acetate buffer (pH 3.2), alkaline picrate; picric acid (0.025 mol L^−1^) in 0.2 mol L^−1^ NaOH, and DNS; dinitrosalicylic acid (2.0% (*w*/*v*)) with phenol (0.5% (*w*/*v*)), and sodium potassium tartrate (0.6 mol L^−1^).

**Table 2:** Summary of recovery studies for the developed CIA method for the simultaneous determination of albumin, creatinine, and glucose in urine.

Sample *^a^*	Albumin Content *^b^* (mg L^−1^, Mean ± SD)			Albumin Recovery (%)	Creatinine Content *^b^* (mg L^−1^, Mean ± SD)			Creatinine Recovery (%)	Glucose Content *^b^* (mg dL^−1^, Mean ± SD)			Glucose Recovery (%)
	Original	Added	Found		Original	Added	Found		Original	Added	Found	
S1 *^c^*	n.d.	30.0	27.0 ± 0.29	89.9	60.5 ± 1.7	100	150.8 ± 2.1	90.3	n.d.	30	25.4 ± 0.2	84.8
S2 *^d^*	n.d.	30.0	32.2 ± 0.41	107.3	57.7 ± 1.4	100	147.8 ± 3.8	90.2	n.d.	30	27.5 ± 0.4	91.7

Note: *^a^* All samples were filtered through a 0.22 µm nylon membrane. *^b^* Determinations were performed in triplicate (*n* = 3). ^*c*,*d*^ Samples were diluted 50- and 100-fold with water, respectively. n.d., not detectable.

**Table 3:** Operational sequence of the analytical procedure for the simultaneous multi-determination of key biomarkers of diabetic nephropathy (albumin, creatinine, and glucose) in urine.

Step	P1	P2	P3	Task Description	Simplified Illustrations of the Steps for the Liquids Handling in the CIA Platforms for the Simultaneous Determination		
					Platform A (*for Albumin*)	Platform B (*for Creatinine*)	Platform C (*for Glucose*)
1	On	Off	Off	Filling the system with carrier (water).	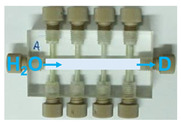	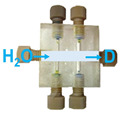	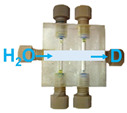
2	On	On (30 Sec.)	On (30 Sec.)	Simultaneous loading of reagents sample, and carrier (Pre-mxing).	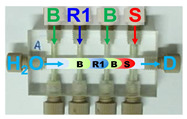	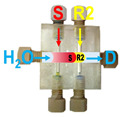	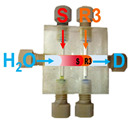
3	On	Off	Off	Delivery of the products to mixing coils and detectors.	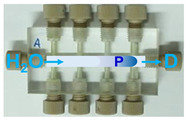	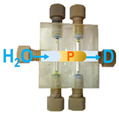	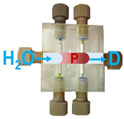
4	On	Off	On (30 Sec.)	Washing the sample and the anlytical flow path conduits.	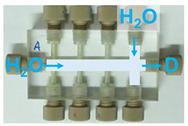	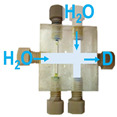	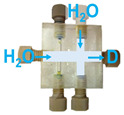

Note: Abbreviations used in Table 3 are defined as follows: D, LED detector; S, urine sample; B, 0.1 mol L^−1^ acetate buffer (pH 3.2); R1, TBPE (2 × 10^−4^ mol L^−1^) with Triton X-100 (0.2% (*v*/*v*)); R2, alkaline picrate (0.025 mol L^−1^ in 0.2 mol L^−1^ NaOH); R3, 2% (*w*/*v*) dinitrosalicylic acid; and, P, developed product zone.

## Data Availability

The original contributions presented in this study are included in the article/Supplementary Material. Further inquiries can be directed to the corresponding author.

## Supplementary Materials

The following supporting information can be downloaded at: https://www.mdpi.com/article/10.3390/molecules31101772/s1, Table S1: Summary of the comparative study on the applications of CIA methods for quantitative analysis; Figure S1: Photographic images of the CIA platforms (including dimensions) employed for the determination of (A) albumin and (B) creatinine and glucose; Figure S2: Absorption spectra of the colored products for the colorimetric detections of standards: (A) albumin (0.1–50 mg L^−1^), (B) creatinine (2.5–25 mg L^−1^), and (C) glucose (100–500 mg L^−1^); Figure S3: Effect of length of mixing coil lengths the sensitivity for the determination of (A) albumin, (B) creatinine, and (C) glucose by the proposed CIA method; Figure S4: Effects of (A) the concentration of DNS and (B) the incubated temperature on the sensitivity of the developed CIA system for the determination of glucose; Table S2: The concentrations of the biomarkers (albumin, creatinine, and glucose) in urine samples, determined by the developed CIA method and by the validation methods; Figure S5: GUI displayed after launching the program; Figure S6: Configuration settings for pump control; Figure S7: Setting the operational status for each step.
