# Supplementary material for: A Novel Cross Injection Analysis for Simultaneous Multi-Determination of Diabetic Nephropathy Biomarkers in Urine
Source: Molecules. 2026 May 21;31(10):1772. doi: 10.3390/molecules31101772 (PMC13209749; doi:10.3390/molecules31101772)
Supplement: Supplementary file 1 [file molecules-31-01772-s001.zip › molecules-4292834-supplementary.pdf]

Supplementary Material

A Novel Cross Injection Analysis for Simultaneous Multi-determination of Diabetic Nephropathy Biomarkers in Urine

Prawpan Inpota [1,2] and Nathawut Choengchan [1,2,*]

[1] Flow-Innovation Research for Science and Technology Laboratories (FIRST Labs), Department of Chemistry, School of Science, King Mongkut's Institute of Technology Ladkrabang, Thailand. praw.inpota@gmail.com (P.I.)

[2] Department of Chemistry and Applied Analytical Chemistry Research Unit, School of Science, King Mongkut's Institute of Technology Ladkrabang, Bangkok 10520, Thailand.

* Correspondence: nathawut.ch@kmitl.ac.th (N.C.), Fax: +66-23298428

This Supplementary information contains four sections (Section S1 to Section S5), including two tables (Tables S1 and S2) and seven figures (Figures S1 to S7)

Section S1: Summary of reported applications of CIA for quantitative analysis.

Table S1. Summary of the comparative study on the applications of CIA methods for quantitative analysis.

No	Methodology / Detection principle	Number of analytes	Sample	[Ref]
1	*'Single'* CIA platform /<br>Colorimetric detection: *o*-phenanthroline method	1 analyte<br>(Total Fe)	Multi-vitamin tablets	[23]
2	*'Dual'* CIA platforms, connected in series as an integrated system /<br>Colorimetric detections:<br>(1) Complex formation with 5-Br-PSAA[a] for Fe^{3+}<br>(2) Jaffé method for creatinine	2 analytes<br>(Fe^{3+} and creatinine)	Human urines	[24]
3	*'Single'* CIA platform /<br>Chemometrics assisted-colorimetric detection:<br>Molybdenum blue method for both PO_4^{3-} and SiO_4^{4-}	2 analytes<br>(PO_4^{3-} and SiO_4^{4-})	Natural waters	[25]
4	*'Triple'* CIA platforms, connected in parallel as an integrated system /<br>Colorimetric detections:<br>(1) ion-association with TBPE[b] for albumin<br>(2) Jaffé method for creatinine<br>(3) non-enzymatic reaction with DNS[c] for glucose	3 analytes<br>(Albumin, creatinine, and glucose)	Human urines	This work

Note: [a] 5-Br-PSAA: 2-(5-bromo-2-pyridylazo)-5-(N-propyl-N-(3-sulfopropyl) amino) aniline, [b] TBPE: Tetrabromophenolphthalein ethyl ester, and [c] DNS: 3,5-dinitrosalicylic acid.

Section S2: Photographic images and configurations of the CIA platforms.

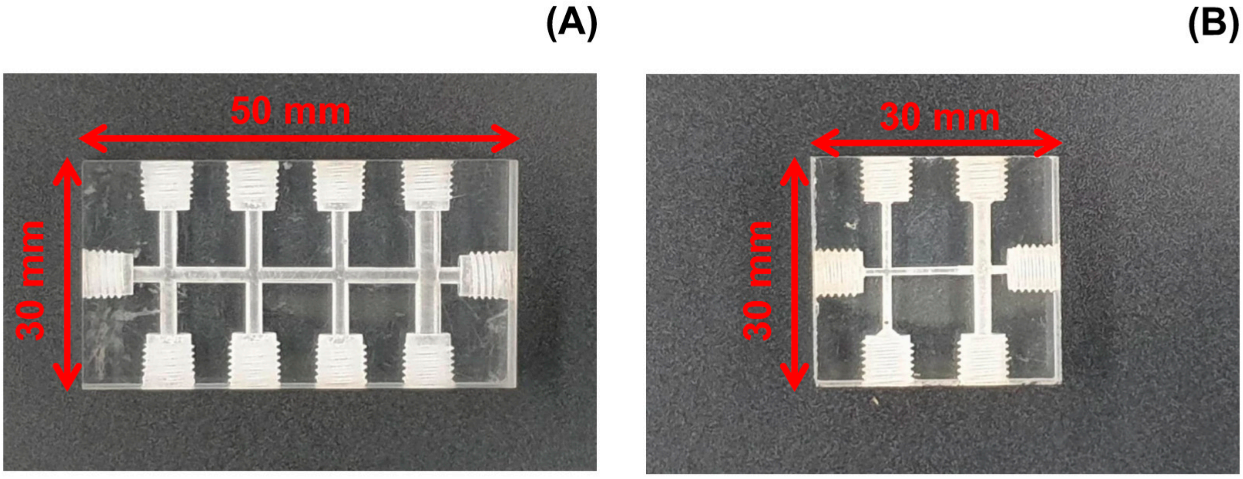

Figure S1. Photographic images of the CIA platforms (including dimensions) employed for the determination of (A) albumin and (B) creatinine and glucose.

\

Section S3: Summary on the optimization study of the CIA system for simultaneous multi-determination of diabetic nephropathy biomarkers.

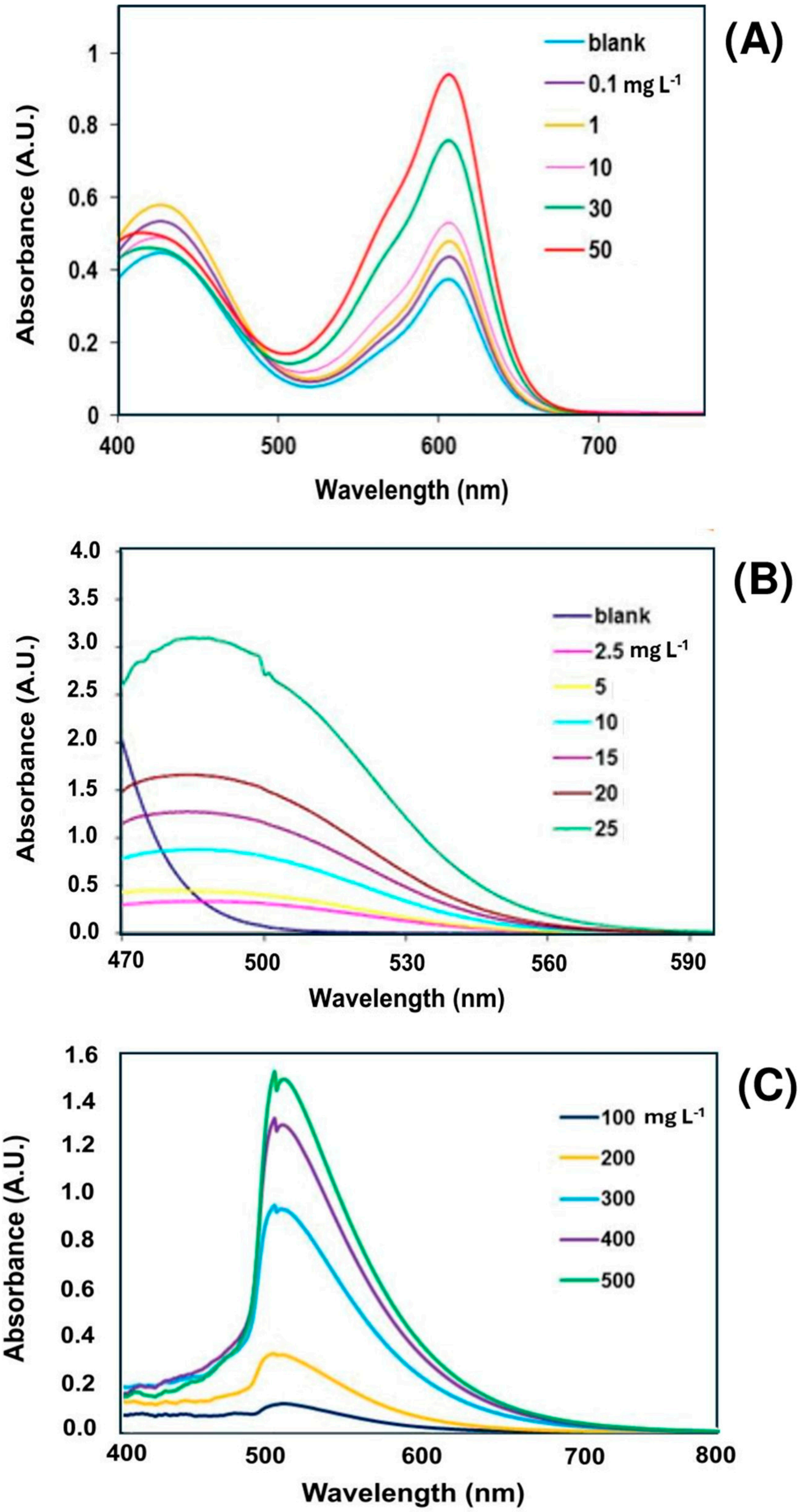

Figure S2. Absorption spectra of the colored products for the colorimetric detections of standards: (A) albumin (0.1-50 mg L^{-1}), (B) creatinine (2.5-25 mg L^{-1}), and (C) glucose (100-500 mg L^{-1}).

Section S3: (Continued)

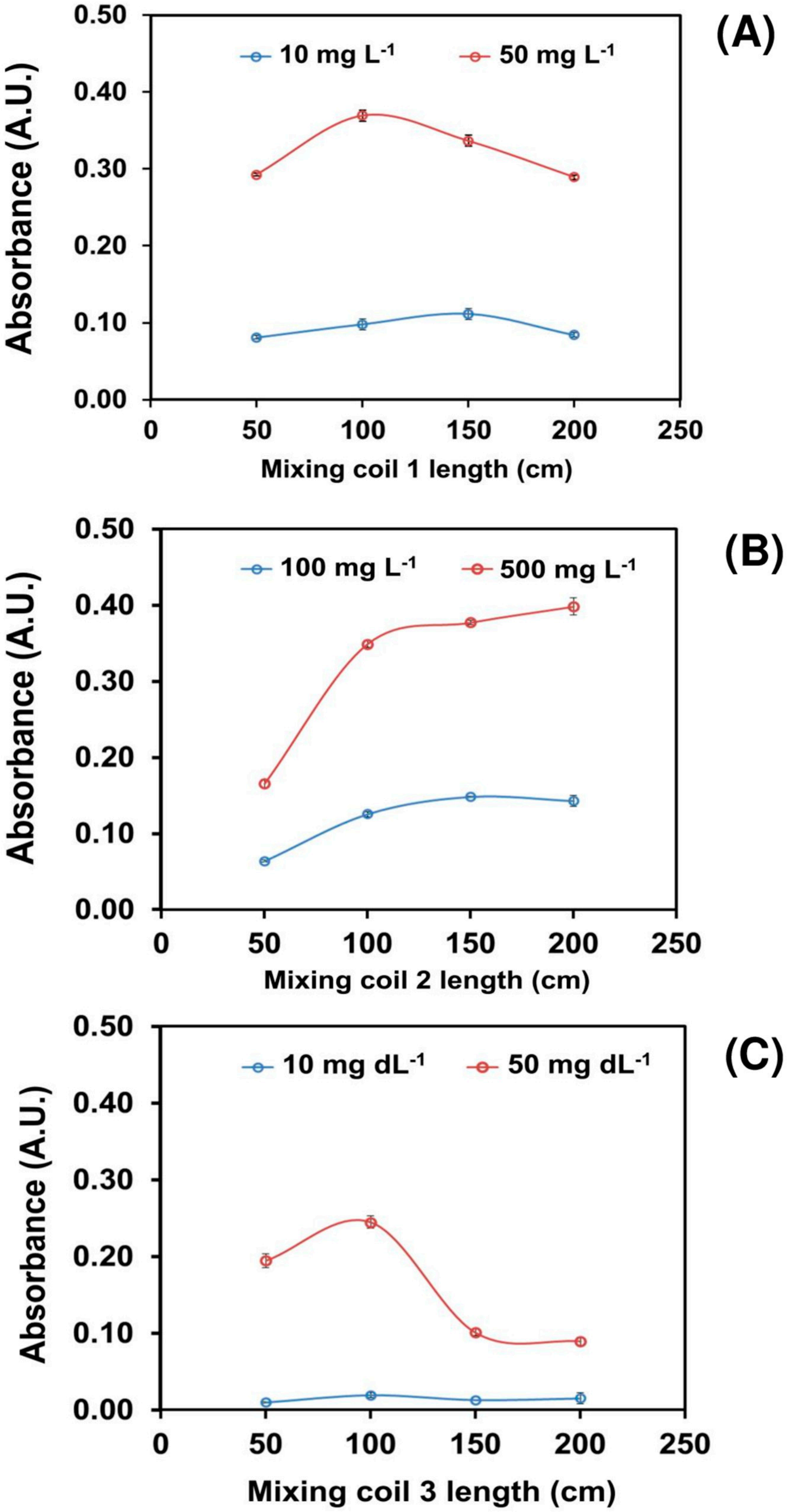

Figure S3. Effect of length of mixing coil lengths the sensitivity for the determination of: (A) albu min, (B) creatinine, and (C) glucose by the proposed CIA method.

Section S3: (Continued)

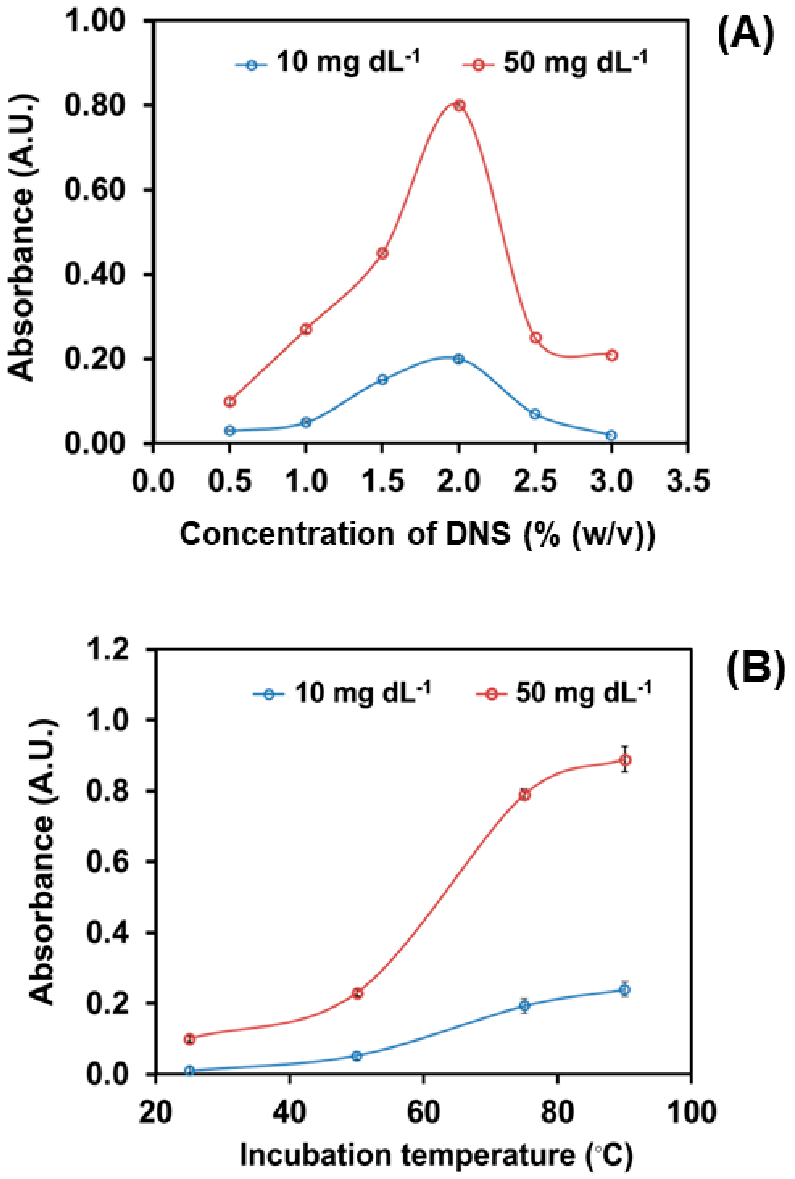

Figure S4. Effect of: (A) the concentration of DNS and (B) the incubated temperature on the sensitivity of the developed CIA system for the determination of glucose.

Section S4: Application to urine samples: Recovery study and validation

Table S2. The concentrations of the biomarkers (albumin, creatinine, and glucose) in urine samples, determined by the developed CIA method and by the validating methods.

Sample	Albumin Content[a] (mg L^{-1}, mean±SD)		Creatinine Content[a] (mg L^{-1}, mean±SD)		Glucose Content[a] (mg dL^{-1}, mean±SD)	
	CIA	Spectrometry[b]	CIA	Spectrometry[c]	CIA	Strip test
S3	14.7 ± 1.9	19.8 ± 3.4	1013 ± 23.1	1178 ± 20.5	n.d.	-
S4	133.9 ± 4.1	140.5 ± 5.6	1425 ± 24.9	1296 ± 22.5	n.d.	-
S5	25.8 ± 2.3	22.1 ± 4.3	782 ± 10.2	900 ± 11.0	n.d.	-
S6	9.0 ± 0.8	6.9 ± 1.1	252 ± 7.2	274 ± 9.6	n.d.	-
S7	215.2 ± 9.2	204.2 ± 6.1	149 ± 6.9	137 ± 5.3	n.d.	-
S8	n.d.	n.d.	101 ± 2.5	104 ± 4.2	234.3 ± 12.1	270 ± 0.0 [d]
S9	n.d.	n.d.	218 ± 8.0	244 ± 5.5	n.d.	-
S10	n.d.	n.d.	161 ± 4.1	118 ± 2.9	n.d.	-

Note: All urine specimens were filtered through a 22-μm membrane filter with subsequent 5 folds with water.

[a] The determination was carried out in triplicate measurements (n = 3).

[b] and [c] Their detection principles are based on the ion-association reaction between albumin and TBPE and the Jaffé reaction, respectively.

[d] The commercial glucose strip test was performed only S8.

Section S5: Operating procedure of the pump control program

1. Open the process control interface developed using Visual Basic 6.0. The graphical user interface (GUI) appears as shown in Figure S5. Prior to operation, the computer must be connected to the PLC controller via an RS-232 serial communication port. The communication port settings can be configured by right-clicking My Computer → Properties → Hardware → Device Manager → Ports (COM & LPT) and setting the parameters as follows:

- COM port = COM1
- Baud rate = 9600
- Data bits = 7
- Parity = Even
- Stop bits = 2

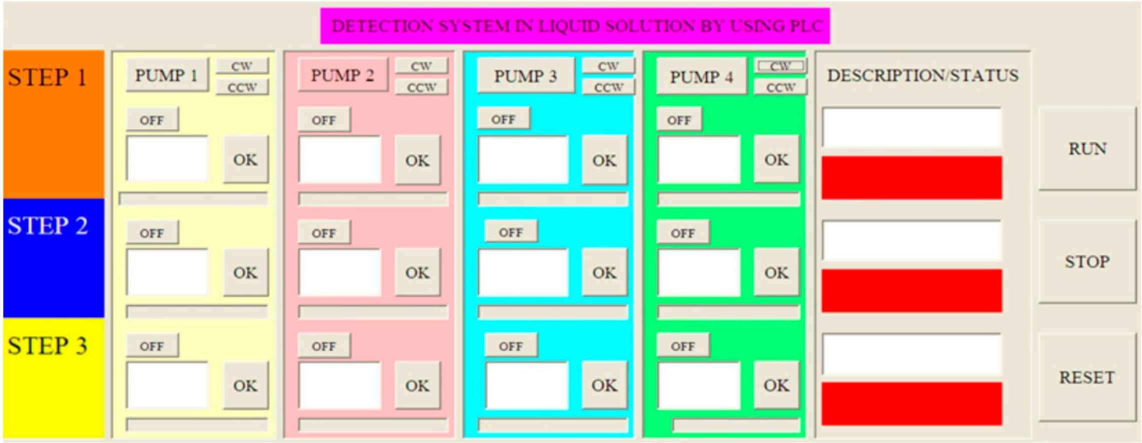

Figure S5. GUI displayed after launching the program.

2. Select the pumps to be operated by clicking the corresponding PUMP buttons.
3. Set the rotation direction for each pump as follows:

- CW = clockwise rotation
- CCW = counterclockwise rotation

4. Configure the operational status of each pump for each process step. The pump can be set either to operate or stop. To activate a pump, click the corresponding button until its status changes to ON. Pumps not required for operation remain in the OFF state, as illustrated in Figure S6.

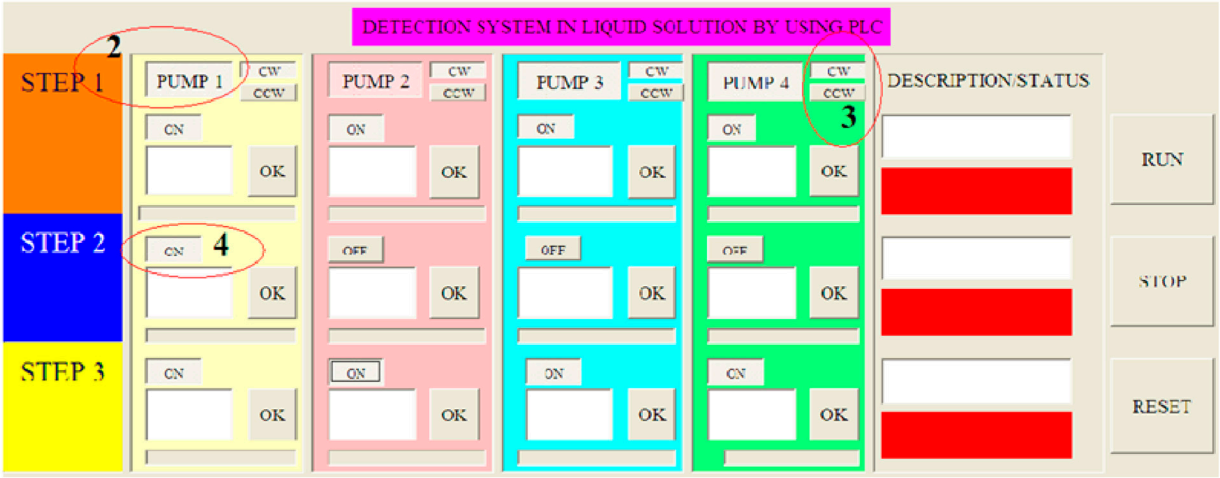

Figure S6. Configuration settings for pump control.

5. Define the operational description for each process step, such as *Loading reagent*, *Detecting*, and *Cleaning*, as shown in Figure S7.

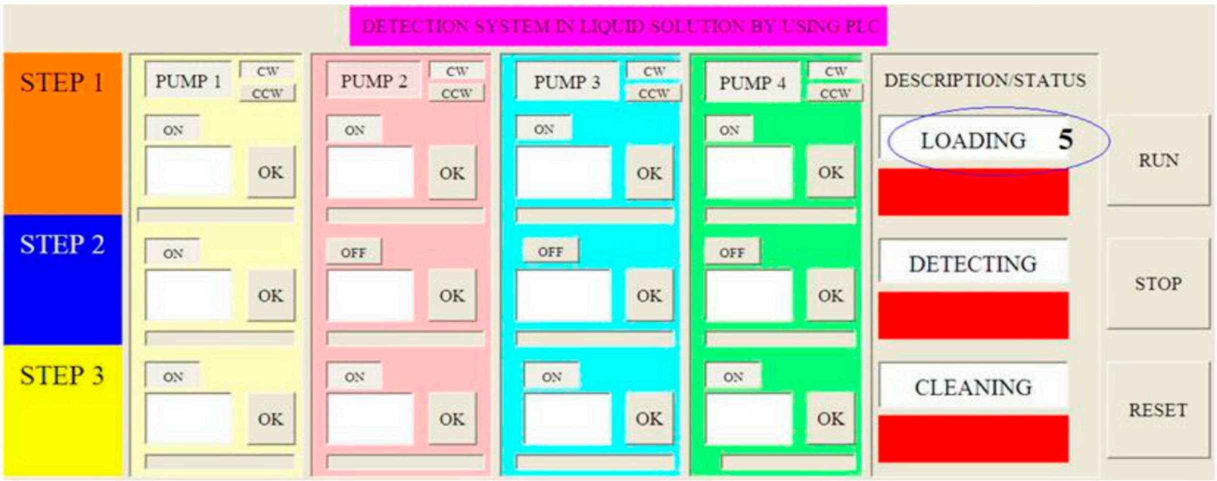

Figure S7. Setting the operational status for each step.

6. Enter the operating time for each pump in each process step. Click the OK button to transfer all parameters to the PLC for storage and processing.
7. Click the RUN button to start system operation.
8. Click the STOP button to terminate all system operations immediately. Once the STOP button has been pressed, the process cannot resume by pressing RUN again.
9. Click the RESET button to clear all operating parameters. This button must be pressed every time the process is terminated, either after pressing STOP or after completion of the operational sequence when Pump 1 finishes its operation.
